# 1.5 million materials narratives generated by chatbots

**DOI:** 10.1038/s41597-024-03886-w

**Published:** 2024-09-28

**Authors:** Yang Jeong Park, Sung Eun Jerng, Sungroh Yoon, Ju Li

**Affiliations:** 1 Massachusetts Institute of Technology, Department of Nuclear Science and Engineering, Cambridge, 02139 USA; 2 Massachusetts Institute of Technology, Department of Materials Science and Engineering, Cambridge, 02139 USA; 3 Seoul National University, Department of Electrical and Computer Engineering, Seoul, 08826 Republic of Korea; 4 The University of Suwon, Department of Environmental and Energy Engineering, Hwaseong-si, 18323 Republic of Korea; 5 Seoul National University, Interdisciplinary Program in Artificial Intelligence, Seoul, 08826 Republic of Korea; 6 Massachusetts Institute of Technology, MIT-IBM Watson AI Lab, Cambridge, 02142 USA

**Keywords:** Computational methods, Solid-state chemistry

## Abstract

The advent of artificial intelligence (AI) has enabled a comprehensive exploration of materials for various applications. However, AI models often prioritize frequently encountered material examples in the scientific literature, limiting the selection of suitable candidates based on inherent physical and chemical attributes. To address this imbalance, we generated a dataset consisting of 1,453,493 natural language-material narratives from OQMD, Materials Project, JARVIS, and AFLOW2 databases based on *ab initio* calculation results that are more evenly distributed across the periodic table. The generated text narratives were then scored by both human experts and GPT-4, based on three rubrics: technical accuracy, language and structure, and relevance and depth of content, showing similar scores but with human-scored depth of content being the most lagging. The integration of multimodal data sources and large language models holds immense potential for AI frameworks to aid the exploration and discovery of solid-state materials for specific applications of interest.

## Background & Summary

Materials are of such significance in human history that the designations assigned to each era of civilization are predicated upon the prevalent materials of the time. With the emergence of the climate crisis, the 21st century has presented humanity with a multitude of challenges, prompting the exploration of novel materials for diverse new applications (solar cells^1,2^, batteries^3–5^, catalysts^6–8^, etc.) in *as short time as possible* in order to wean the *entire economy* off burning fossil fuels. The expeditious discovery of materials possessing desirable attributes for specific applications garners considerable attention; however, it is impeded by the lack of digestible information (to a mechanical or electrical engineer, for example) about materials. For example, when asked about a specific material “Li_4_Mn_5_Ni(PO_4_)_6_”, even a materials expert would usually turn to Google search, and the outcome would likely be quite dense and varied literature with no guarantee of finding what one wants, that can take hours or days to parse through, which is just too slow, especially if all one needs is an initial screening. Oftentimes, it is hard to present aggregated information, as properties are spread over multiple experimental and *ab initio* databases.

The desired attributes (figure-of-merit) required to realize a given specific device may be known, while the specific materials embodying superior figure-of-merit are generally unknown and more difficult to identify. Throughout history, materials with technological functionalities have frequently been discovered through a combination of intuition, trial and error, and fortuitous circumstances. Today, the prevailing paradigm has transitioned towards a more comprehensive exploration of the vast space of potential materials. This endeavor is facilitated by the applications of first-principles calculations and artificial intelligence (AI). Notably, the advent of generative AI models has spurred a surge of research into the realm of inverse material design^9–11^. Through the utilization of generative AI techniques, researchers have been able to accelerate the process of materials discovery and design, offering promising opportunities for breakthroughs in the figure-of-merit for specific applications. Some of the authors have also examined the utilization of automated systems capable of generating scientific hypotheses in their recent work^12^. These systems based on large language model (LLM), including chatbots such as ChatGPT^13^, possess an inherent probabilistic nature that enables them to generate intriguing hypotheses, thereby expediting scientific advancements akin to human researchers. However, the examples presented in the Supplementary Information section 1 also demonstrate certain challenges with the “common-core” LLMs such as the standard ChatGPT, including bias toward “hot materials” and “hot topics”, whereas true ground-breaking innovations may spring from “cold topics” or less well-known materials^12^. The “common-core” LLMs, owing to their learning process based on the probabilistic distribution of tokens, tend to prioritize the presentation of materials frequently encountered on the web and in scientific literature and publications^14–18^, rather than “comprehending” the inherent properties and structures of materials and selecting suitable candidates more rationally. This is because the “common-core” text corpora found on the web are highly tilted toward materials already studied by human researchers, which can be rather limited, as researchers tend to flock toward “hot materials”. This may limit the inventiveness of the narratives and inferences generated directly with “common-core” ChatGPT^12^. The present work aims to generate more balanced plain-language materials narratives that can be supplemented to the common corpus and used to *further* train more specialized LLMs so their inferences will be less biased toward “hot” but narrow-based materials.

In recent years substantial progress has been made in the realm of multimodal learning across diverse domains. The amalgamation and integration of information from various modalities, encompassing text, images, audio, and video, have facilitated breakthroughs in comprehending intricate data. This interdisciplinary approach has yielded remarkable applications in computer vision, natural language processing (NLP), and audio analysis, thus empowering the development of more comprehensive and resilient learning systems. However, the field of materials research has yet to embrace the endeavor of multimodal learning. To surmount these challenges, our research team has generated and shared data of 1,453,493 natural language-material pairs utilizing publicly available material databases and chatbots. This is a fairly large number considering that the number of training images in ImageNet is 1,281,167.

The fusion and convergence of multiple modalities to enhance learning and comprehension of materials represent relatively uncharted territory. However, given the rapid advancements in machine learning and the increasing availability of multimodal datasets, this captivating area of study harbors considerable potential for future research and innovation. Our textual narratives will serve as an initial stepping stone towards pioneering novel subfields of AI, such as materials captioning, materials multimodal learning, and simulation automation.

## Methods

### Materials imbalances in common corpus

**Fig. 1:**
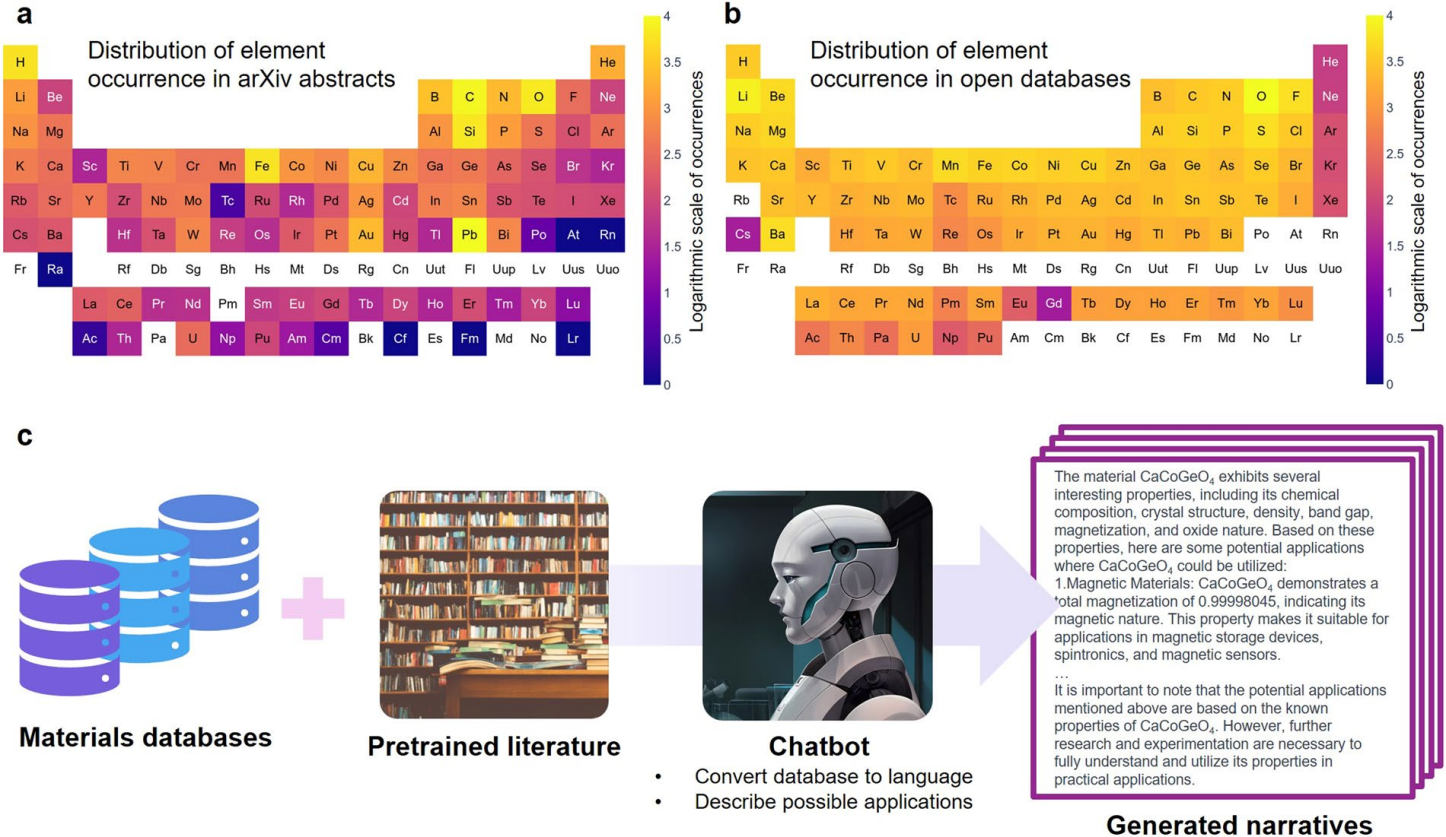
Overall data synthesis framework proposed in this work. (**a**) Distribution of chemical elements invoked in materials studied in the materials research literature. (**b**) Distribution of chemical elements in publicly accessible databases that are mostly generated by *ab initio* calculations. (**c**) The proposed framework that extracts knowledge about materials science to overcome the discrepancy between the materials studied in research and those available in public databases.

We visualize the bias present in the distribution of materials described in the common-core text corpus, which for ChatGPT^13^ are array of sources available on the internet prior to September 2021. This includes a diverse range of documents, websites, books, and other text-based sources. To identify patterns of material bias found in actual academic literature, we utilized the arXiv dataset hosted by the joint automated repository for various integrated simulations (JARVIS)^19^. Specifically, we selected abstracts from 284,815 papers in the ‘cond-mat’ category. In order to identify the frequency of appearance of a material, as the chemical space is rapidly enlarged when a material of more than binary elements is included, the frequency of occurrence was extracted by searching for a matching pattern using a regular expression for each element. We then extracted and visualized the occurrence frequencies by searching for matching patterns using regular expressions for each element. At the same time, the appearance frequencies of elements included in materials stored in publicly accessible databases such as Materials Projects^20^, JARVIS^19^, and Open Quantum Material Database (OQMD)^21^ were extracted and visualized. As shown in Fig. 1a,b, the materials studied within the research community focus on oxides, with a high occurrence frequency of familiar materials such as iron and copper. In contrast, most chemical elements (excluding noble gases) are much more evenly distributed in materials addressed by *ab initio* databases. The graph illustrates the bias or imbalance in materials of interest in a), focusing on oxides and frequently encountered materials like iron and copper. In contrast, the distribution of materials in b), excluding noble gases, is more evenly distributed, in open databases such as Materials Projects, JARVIS, and OQMD. This means that if we could combine the knowledge presented in specialized *ab initio* databases with a “common-core” LLM^12^, we could produce more balanced narratives that can be used to *further* train more specialized LLMs so their inferences will be less biased toward “hot” but narrow-based materials (Fig. 1c). With such more specialized LLMs, we could extrapolate trained information of language models from the scientific literature. For instance, a language model can extract the fact that a material possessing an appropriate bandgap, electrical conductivity, and stability can be considered a potential semiconductor candidate.

### Material narrative text generation

**Fig. 2:**
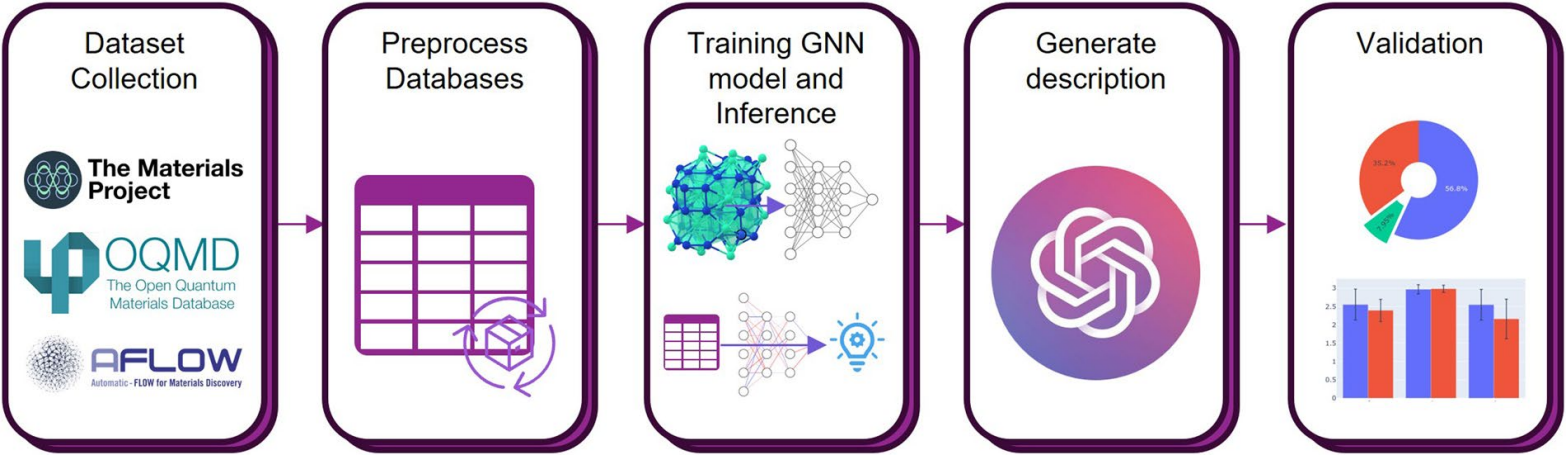
Summary of the materials narrative generation process. The pipeline involved data collection from the joint automated repository for various integrated simulations (JARVIS). The databases were preprocessed to select relevant properties for textual narrative generation. A crystal graph neural network (GNN) model was trained to extrapolate properties across multiple databases. The generated narrative went through two stages: first, converting the data into a dictionary and requesting a description, and second, using the generated result to obtain the final narrative. The generated narratives were evaluated by human experts and GPT-4, and a validation process was conducted to evaluate correctness and detect potential adverse effects.

The process of generating the narrative of materials is summarized in Fig. 2. Data collection pipeline was mainly implemented using Python programming language (version 3.9.15) and PyTorch^22^ (version 2.0.0), widely used in deep learning. All the computations were performed on a high-performance workstation with specifications including Intel® Core™ i9-10920X X-series Processor and NVIDIA RTX3090 graphic processing units (GPUs).

#### Data collection

We obtained material data from publicly available repositories, the JARVIS^19^. The dataset encompassed diverse materials and covered a wide range of density functional theory (DFT) calculated properties. Moreover, JARVIS also provides an integrated way to access other publicly available databases such as Materials Projects and OQMD. The selected databases and number of materials included are described in Table 1.

**Table 1:** Number of materials used in this work.

Database name	Number of materials
JARVIS	55,723
Materials Project	126,335
OQMD	851,300
Aflow2	420,135
Total	1,453,493

#### Preprocessing databases

To generate textual narratives, some of the properties provided by each published database were heuristically selected. For example, scalar physical quantities such as “band gap” and “formation energy per atom”, categorical data such as “crystal system”, and Boolean data such as “stable” were mainly selected. The number of materials for which the properties were provided for each open database is summarized and shown in Table 2. The types of attributes provided are inconsistent and the number of types is different. For example, some properties such as “band gap” and “formation energy per atom” are provided by several databases, but some properties such as scintillation attenuation length are only provided by AFLOW2.

**Table 2:** Property prediction performance metrics of graph neural network models.

Property	Units	Dataset	Number	MAD	MAE	MAD:MAE
Total energy per atom	eV/atom	OQMD	312,675	1.642	0.06307	26.03
Formation energy per atom	eV/atom	OQMD	312,670	0.6634	0.04511	14.71
Energy above hull	eV/atom	Materials Project	126,335	0.2055	0.0501	4.104
Band gap	eV	Materials Project	126,335	1.233	0.2484	4.963
Enthalpy per atom	eV/atom	AFlow2	420,135	1.732	0.0307	56.36
Scintillation attenuation length	cm	AFlow2	420,135	0.8242	0.0186	44.36

The trained model was employed to extrapolate properties using materials from multiple databases as input.

#### Training GNN model and inference

Inconsistencies in attributes provided between databases can harm the uniformity of the generated data. For example, in a database that only provides a band gap, it may be difficult to create a meaningful narrative because of insufficient context for the material. Therefore, it was extrapolated using a graph deep learning model to create narratives with a similar number of attributes regardless of the source database. The model was modified to be E(3) equivariant based on ALIGNN^23^, which was successful in predicting quantum chemical properties.

The selected GNN model was implemented using deep learning frameworks, PyTorch and Deep Graph Library (DGL)^24^. The AdamW optimizer with normalized weight decay of 10^−5^ was used. A learning rate reduction strategy during plateaus was employed and training was conducted for 500 epochs with early stopping applied if no improvement was observed. The model was trained on high-performance computing systems equipped with powerful GPUs. The trained model was used to extrapolate each property with materials from multiple databases as input. The training results for each model are in Table 2. To evaluate the accuracy of the model’s predictions, the MAD:MAE ratio was used^23^. A higher ratio indicates that the model’s prediction error is small compared to the inherent variability of the data. Training a language model using narratives synthesized from property values predicted by a less predictive model can introduce significant confusion. To prevent this, we excluded properties with a MAD:MAE ratio of 4 or less.

#### Generating narratives

Creating the narrative was done in two stages. First, the data frame obtained by extrapolation was converted into a dictionary and requested as follows.

“*The following dictionary contains the composition and properties of a material stored in the database. Please write a description of the material, referring to this information. Make sure not to omit any item, and include all numerical values, citing their units appropriately. Feel free to include brief explanations or qualitative meanings for each property*.”+ *dictionary of given material*

After that, the generated result was used as input again to obtain a final narrative.

“*Let’s assume that we have a material with the following properties. Provide possible application areas for this material and explain the rationale behind them*.”+ *generated text*

This format shows a similar tendency to report new materials in academic papers. It is meaningful to follow a similar format as most researchers report the properties of a new material first and then list possible applications from it. As a result, the average token length of the dataset is 788.6, with the longest being 1,585 tokens. This makes it suitable for fine-tuning models with a context length of 2,048, including custom instruction.

#### Validation

Evaluate whether the resulting material narrative is correctly described and free of other potential adverse effects. All narratives were generated with GPT-3.5-turbo (GPT-3.5) and evaluated by human experts and GPT-4. In addition, it was investigated whether it was possible to identify whether the generated contents were written by generative AI. A more detailed process is described in the Technical Validation Section.

## Data Records

The 1.5 million pieces of natural language-material narratives generated via a chatbot in this work are deposited on HuggingFace Datasets^25^. At HuggingFace, various NLP data, model weights, and training tools are provided, and continuous data maintenance is supported through the Git version control system with contributions from the community. The database is organized in Apache Parquet^26^ format where elements in each column represent the same contents such as chemical properties, chemical formula, or generated text, and elements in the same row relate to the same material.

## Technical Validation

Like any narrative from any source, ours will also contain factual errors and soft inaccuracies. The key is to reduce these as much as possible.

**Fig. 3:**
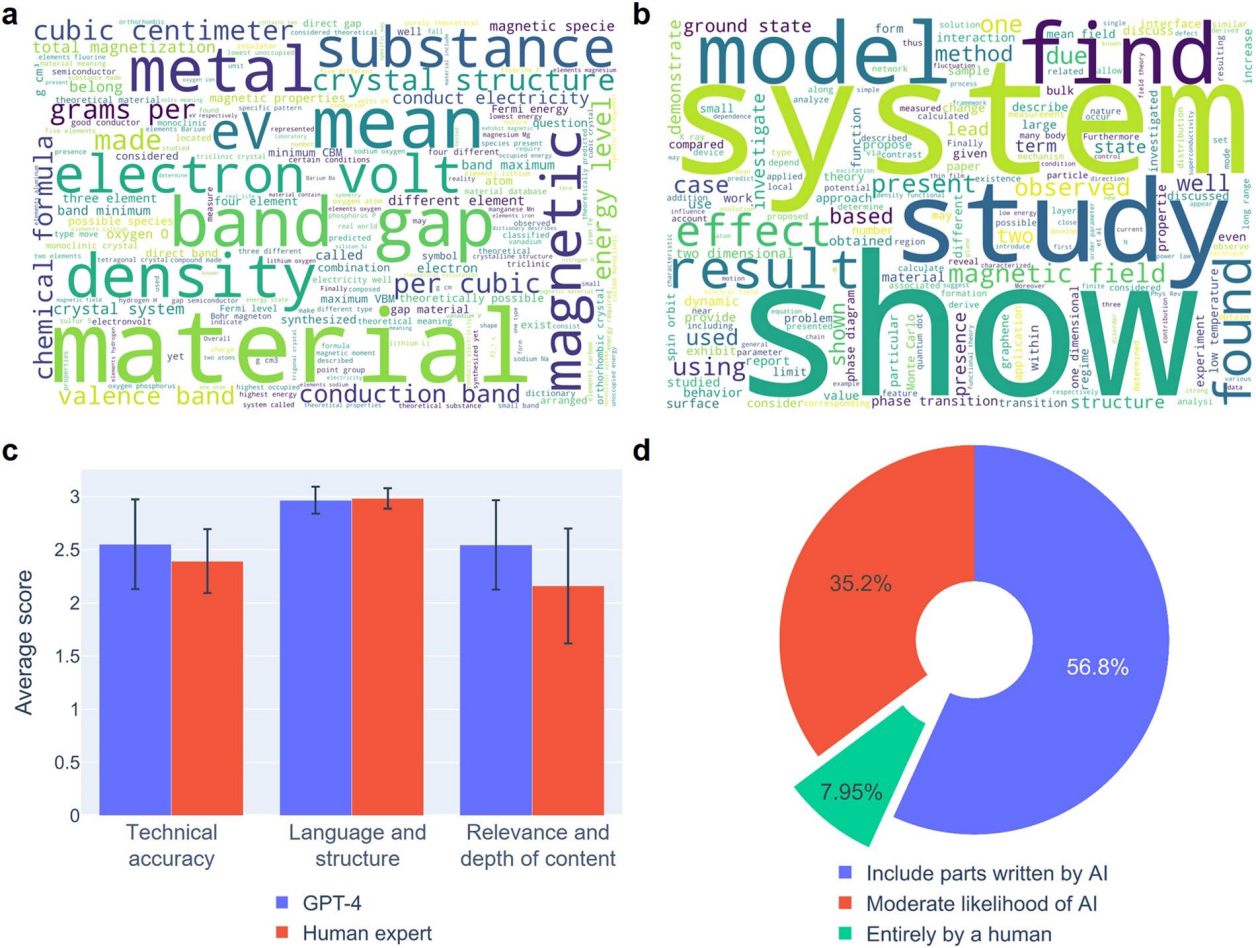
Details of the generated narratives (**a**) The word cloud visualization shows the highlighted words in the JARVIS-arXiv dataset, indicating the materials studied and their specific results. (**b**) The word cloud visualization of narratives generated from databases, often including possible applications based on stored material properties. (**c**) Evaluation results of the randomly sampled 1,067 generated narratives evaluated by both human experts and GPT-4. (**d**) GPTZero^32^ classification results of generated narratives to address concerns of data contamination, achieving over 92% accuracy in classifying the generated text.

The quality of the text generated by the word cloud visualization was evaluated in Fig. 3a,b. A word cloud is a visual indicator of the frequency and importance of text, helping us to identify key themes and emphasized words in the whole text. Through this, it was possible to evaluate how diverse and meaningful the generated texts were and how faithful they were to the main theme. In the JARVIS-arXiv dataset, all the input text is abstract, so the corresponding word is highlighted to indicate that the material was studied and produced a specific result. In common, since each material in the generated narrative shows an almost uniform element distribution, it has a relatively low frequency of appearance, so it is not visualized in the word cloud. On the other hand, narratives generated from databases are often visualized with descriptions of possible applications based on the stored material properties.

It is impractical to manually validate the hundreds of thousands of generated sentences individually. Since GPT-3.5 has already demonstrated its ability to generate natural-sounding sentences, traditional metrics used in NLP such as BLEU^27^, ROUGE^28^, and perplexity scores, which quantify similarity, coherence, and fluency of generated sentences, are not suitable for evaluating scientific and academic writing. Recently, a method to evaluate weak LLM using strong LLM as an evaluator was proposed^29,30^. Studies have shown that human preference and GPT-4 show 80% agreement. This means that the reason- ing power of LLM can be used to automate large-scale evaluation tasks that would be impossible for humans, such as our dataset. Therefore, we automated the evaluation using GPT-4 based on the following rubrics (Fig. 3c). GPT-4 and human experts are asked to critique the narratives based on the following prompt: “You are a materials scientist. Please critique the following description and assign a rating of up to three stars based on the following three rubrics. <Rubrics below> <Narrative>”.

### Technical accuracy

The first and most crucial step is to evaluate the factual accuracy of the article. As the article is related to material science, it should properly represent scientific theories, facts, experimental observations, and material properties.

### Language and structure

This evaluates how the AI has organized and presented the information. Is the article logically structured? Are the sentences well-formed and free of grammatical errors? Does the language use meet the standard of a scientific paper or article? The language should be clear and precise, and the information should be organized in a coherent and easy-to-follow manner.

### Relevance and depth of content

This step examines whether the AI-generated content stays on topic and goes into enough depth. It should not merely scratch the surface of the subject but delve into the complexities and nuances. Also, the AI should not drift away from the topic or include irrelevant information.

To ensure statistical representativeness, we randomly selected 1,067 narratives from the 1.5 million narratives generated using a random seed of 42 for evaluation. This selection accounts for a margin of error of plus or minus 3 percent at a 95 percent level of confidence. The selected texts were evaluated and compared by human experts as well as GPT-4. To perform evaluations and compare them on the same rubrics, human annotators received the same instructions as GPT-4. The evaluation results showed similar results in human experts and GPT-4. The texts are well organized, based on the database, and grammatically and structurally almost perfect. However, it is noteworthy that the human expert group gave a rather low score for the depth of the content.

Contamination of content created using generative AI by mixing it with the original content is one of the challenges facing the large language model (LLM) community^31,32^. It is important to recognize the risks that the textual narratives generated by our method will be distributed indiscriminately as “100% factual” and get mixed with human-generated text, polluting the corpus and hindering the progress of science and technology. In this context, various sensing technologies have emerged to prevent contamination and prevent indiscriminate usage. We used GPTZero^32^, one of the important early contributions to deep learning security for detecting AI-generated text^33–35^, to assess the risk of our generated text going undetected. The results in Fig. 3d were correctly classified as over 92% AI-written text, mitigating the risk somewhat, but still such risk is present.

## Usage Notes

The natural language text-material narratives created here can serve as a new starting point for LLM-based inverse material design to discover functional materials *in silico*, linking the efforts of the NLP and materials science communities. Examples of possible approaches for inverse engineering techniques using this database are as follows:

1. Language-crystal multimodal learning and inference of materials. By using NLP, it is possible to identify a subset of initial material structures with desired characteristics and desired application fields and convert it into actual first-principles calculation input through tools such as pymatgen^36^ and ASE^37^.
2. Fine-tuning LLMs for scientific purposes. It is expected that large-scale applications of scientific hypothesis machines^12^ can be achieved by fine-tuning large language models for specific purposes, based on domain-specific databases.
3. Vector database and use for in-context learning. Due to the emergent abilities^38^ of LLMs, it is expected that vocabulary used in material science fields can be understood “naturally” if a sufficiently large model is used.

Also, it is important to quantify the current level of understanding of the material of LLMs. By achieving a higher level of understanding, we can reduce dependency on external databases and reduce computational costs. We propose two metrics.

### Materials to properties (Mat2Props)

Understanding the physical and chemical properties of materials using an LLM could be useful for future AI systems. This is distinct from simply using external data or functions to return an exact value. If the LLM itself can independently predict multiple properties of given materials, it can activate various downstream tasks using the inherent inference path within the LLM. This is similar to why we need to learn mathematics even though calculators exist in modern times. GPT-3.5 is asked to predict the multiple properties of a given material simultaneously (Table 3). Depending on the purpose, inference can be performed using the chemical formula of the material, or by using a crystallographic information file (CIF) as input. Developing these abilities is potentially related to hallucinations. Reducing hallucinations allows the model to attempt to retrieve stored property values from implicit knowledge instead of inventing plausible numbers. We found that high performance can be achieved by constructing a retrieval-augmented generation (RAG)^39^ combined with an external database, but we expect that a high level of generalization can also be achieved by reducing the hallucination of the model.

**Table 3:** Property prediction performance of machine learning models on the Materials Project dataset^23^.

Prop	Unit	MAD	CFID	CGCNN	MEGNet	SchNet	ALIGNN	GPT-3.5
Formation energy (*E*_*f*_)	eV/at.	0.93	0.104	0.039	0.028	0.035	0.022	1.897
Band gap (*E*_*g*_)	eV	1.35	0.434	0.388	0.33	—	0.218	1.309

Unlike other GNN models, GPT-3.5 was asked to predict all properties at once. All performances were measured using MAE, except for MAD.

### Materials to multiple-choice questions (Mat2MCQ)

**Fig. 4:**
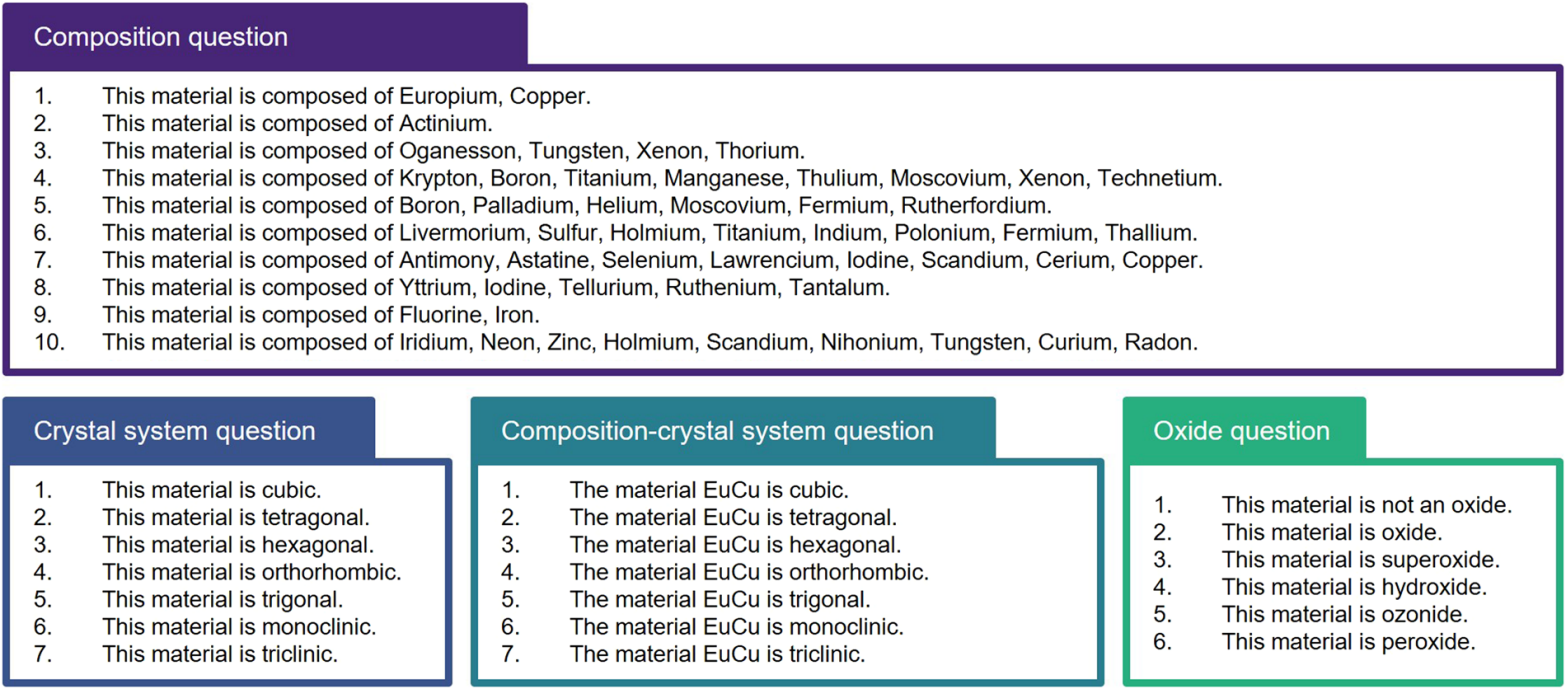
An example of multiple-choice questions for the copper europium. It is designed to evaluate whether an AI model can capture the elements and structural characteristics contained in the given material. Such questionnaires can be easily created using random numbers and expanded similarly.

**Fig. 5:**
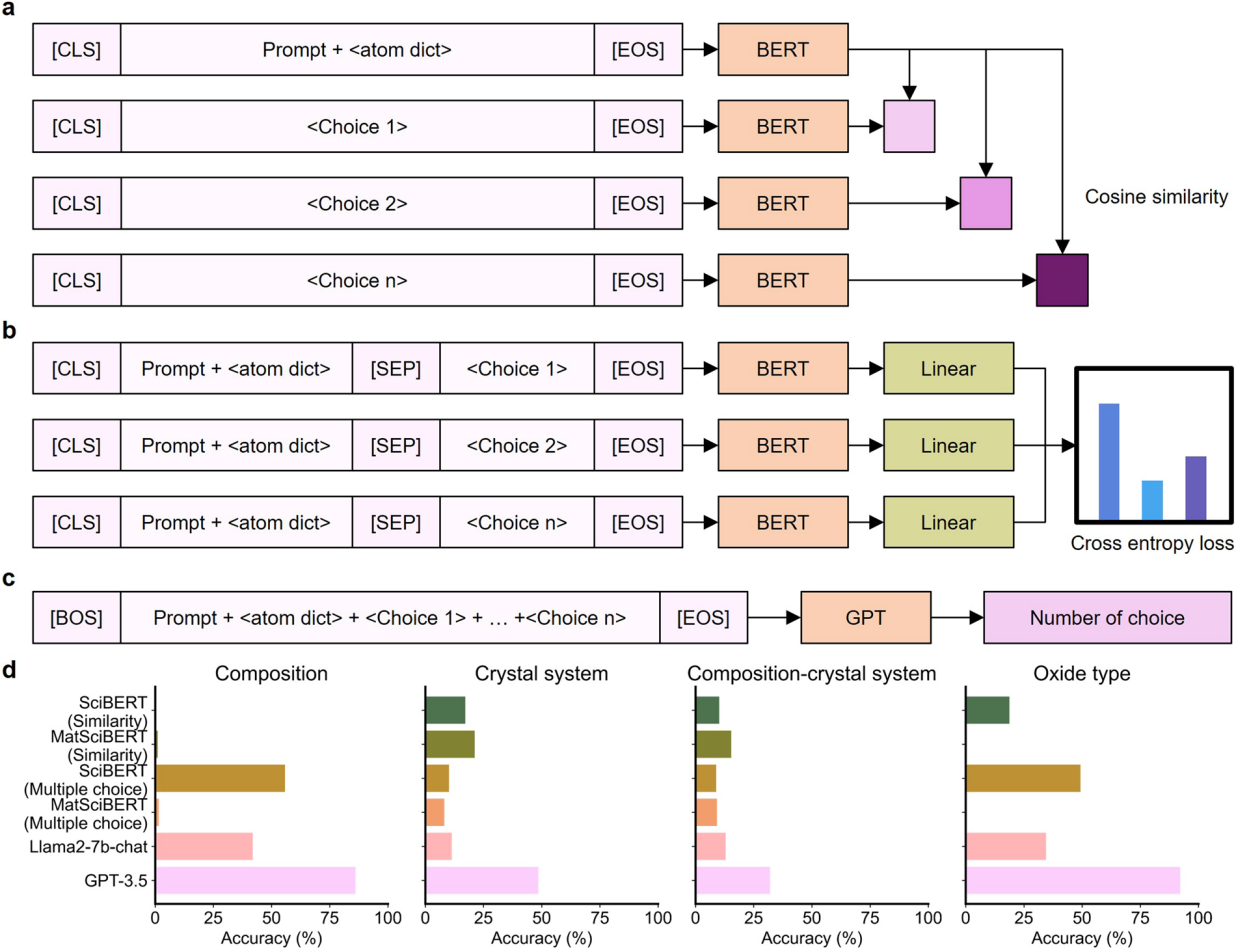
Evaluation of baseline models for multiple-choice question answering for materials. (**a**) The input consists of a [CLS] token, the prompt concatenated with an atomic dictionary (atom dict), and an [EOS] token. Each choice is separately encoded to determine the similarity with prompt. (**b**) The prompt is concatenated with the atom dictionary and each choice is combined into a single input sequence separated by [SEP] tokens. Linear layers are applied to the outputs to generate scores for each choice, followed by comparison to select the best answer. (**c**) The prompt, atom dictionary, and all choices are concatenated into a single sequence, starting with a [BOS] token and ending with an [EOS] token. The decoder-only transformer determines the most likely answer. (**d**) The performance of each approach at multiple-choice question answering tasks.

Prediction of containing elements or crystal structure from a given material helps improve the overall understanding of the material. We propose multiple-choice questions to assess this understanding. An example of this problem is shown in Fig. 4. There may be several approaches to solving this problem. Here we present three baseline approaches in Fig. 5. The first approach is to compare the similarity between the embedding from the given material and the embedding of choices. Encoder-only LLMs such as SciBERT^40^ and MatSciBERT^41^ were evaluated by this method. The second method involves predicting the similarity between prompt and choice connected by a [SEP] token. This is achieved using a linear layer followed by a softmax function to predict the most correct answer. Encoder-only LLMs were also evaluated using this method. The third approach uses a decoder-only generative model. CIF format is not suitable since it contains information about the crystal system and its long token length. Instead, we use the dictionary format which includes atoms and unit cell information provided by JARVIS. The dictionary representation of the given material and all choices are presented simultaneously in a prompt that is written to select the correct answer. If creation fails, it is considered a wrong answer. To ensure a fair comparison, all models performed zero-shot inference. The performance of the popular transformer models such as GPT-3.5 and Llama-7b-chat^42^ models is shown in Fig. 5d. An evaluation was conducted on the 10% of the Materials project. Although this information may seem simple, it turns out to still be challenging even for the latest open-source LLMs. In particular, even GPT-3.5 did not achieve a high score on the problem of classifying crystal systems. Mat2MCQ also can be used to develop better text representations of materials.

We believe that the intelligence-driven “fifth paradigm”^43,44^ of material discovery can be further advanced by introducing an approach that goes beyond simply providing data. Rather than relying solely on connections to external databases, chatbots that can learn from an expanded material space, identify inherent patterns, and express them in human language will become a form of explainable AI for materials science. Additionally, as the shortage of data to train AI models becomes a reality, we believe that synthetic data approaches are a promising way to push the boundaries.

## Supplementary information

Supplementary Information

## Data Availability

Code for reproducing this work is available in the GitHub repository (https://github.com/parkyjmit/GPT-Narratives-for-Materials).
