# Supplementary material for: 1.5 million materials narratives generated by chatbots
Source: Sci Data. 2024 Sep 28;11:1060. doi: 10.1038/s41597-024-03886-w (PMC11439064; doi:10.1038/s41597-024-03886-w)
Supplement: Supplementary file 1 — Supplementary Information [file 41597_2024_3886_MOESM1_ESM.pdf]

Supporting Information

1 Limitations of Causal Language Models in Material Selection

In this section, we asked GPT-3.5 to create solid electrolyte materials for potential all-solid-state batteries:

'You are a great materials scientist. Please recommend potential solid electrolyte materials for the development of new all-solid-state batteries.'

The question was repeated three times, and a new agent was called every time instead of reusing the answer to prevent context contamination. Although the answers generated were different due to the stochastic nature of the generative model, it was found that certain materials appeared consistently. Among the commonly mentioned materials are: lithium garnet, such as Li7La3Zr2O12 (LLZO)[14,15], perovskite-type oxides[16], Sodium Super-Ionic Conductors (NASICON) materials, such as Na3Zr2Si2PO12 (NZSP) and NZPSO[17], or Sulfide-based materials like LGPS and LPS[18].

It is important to note that the responses may not be exhaustive, and the field of solid-state batteries is evolving, with ongoing research introducing new materials and advancements. Staying updated with the latest scientific literature is crucial to keep track of the most recent developments in the field of all-solid-state batteries.

Q1: You are a great materials scientist. Please recommend potential solid electrolyte materials for the development of new all-solid-state batteries.

A1: Thank you for the compliment! Solid-state batteries are an exciting area of research, and there are several potential solid electrolyte materials that show promise for their development. Here are a few recommendations:

1. Lithium Garnet (Li7La3Zr2O12 or LLZO): Lithium garnet is a popular solid electrolyte material due to its high ionic conductivity and stability with lithium metal electrodes. It has a wide electrochemical stability window and exhibits good mechanical properties. LLZO has been extensively studied and shows promise for high-performance solid-state batteries.

2. Perovskite-Type Oxides: Perovskite-type oxides, such as LaAlO3, LaGaO3, and Li0.33La0.557TiO3, have attracted attention as solid electrolyte materials. They exhibit high ionic conductivity and good stability with lithium. However, they may suffer from limited mechanical stability, requiring additional optimization and stabilization strategies.

3. NASICON-Type Materials: Sodium Super-Ionic Conductors (NASICON) materials, like Na3Zr2Si2PO12 (NZSP), have been investigated for solid-state batteries. They offer good ionic conductivity, stability, and are more abundant and less expensive compared to lithium-based materials. Sodium-ion solid-state batteries utilizing NASICON-type materials show promise for future energy storage applications.

4. Sulfide-Based Materials: Sulfide-based materials, such as Li10GeP2S12 (LGPS) and Li7P3S11, have shown high ionic conductivity and good stability with lithium electrodes. Sulfide-based solid electrolytes typically have higher ionic conductivity than oxide-based materials but may require careful handling due to their air and moisture sensitivity.

5. Composite and Hybrid Materials: Composite and hybrid solid electrolyte materials can combine different components to enhance overall performance. For example, a composite of LLZO and a polymer matrix can provide both high ionic conductivity and improved mechanical properties. Hybrid electrolytes, combining solid and liquid components, can also offer high conductivity while maintaining improved safety.

It's worth noting that the field of solid-state batteries is rapidly evolving, and ongoing research may introduce new materials or advancements. Therefore, it's essential to stay up-to-date with the latest scientific literature and technological developments in this area.

Q2: You are a great materials scientist. Please recommend potential solid electrolyte materials for the development of new all-solid-state batteries.

A2: Thank you for your kind words! All-solid-state batteries are a promising technology for next-generation energy storage. They offer several advantages over traditional liquid electrolyte batteries, such as improved safety, higher energy density, and wider operating temperature range. In the development of all-solid-state batteries, the choice of solid electrolyte materials is crucial. Here are a few potential solid electrolyte materials that have shown promise:

Lithium Garnet-Type Materials: Lithium garnet materials, such as Li7La3Zr2O12 (LLZO), Li6.25Al0.25La3Zr2O12 (LLAZ), and Li6.75La3Zr1.75Ta0.25O12 (LLZTO), are widely studied for all-solid-state batteries. They exhibit excellent chemical and electrochemical stability, high ionic conductivity, and good compatibility with lithium metal anodes.

Perovskite-Type Materials: Perovskite oxides, including various compositions of ABO3, have attracted attention as solid electrolytes. Examples include La0.5Li0.5TiO3 (LLTO), Li3xLa2/3-xTiO3 (LLTO), and BaZrO3-based materials. Perovskite electrolytes often exhibit high ionic conductivity at elevated temperatures and can be doped or modified to enhance their performance.

Sulfide-Based Materials: Sulfide-based electrolytes, such as Li10GeP2S12 (LGPS), Li7P3S11 (LPS), and Li3PS4 (LPSI), have demonstrated high ionic conductivity at room temperature. They offer good compatibility with lithium metal and can operate at lower temperatures compared to oxide-based electrolytes. However, sulfide electrolytes may have challenges related to stability and compatibility with other cell components.

NASICON-Type Materials: Sodium superionic conductor (NASICON) materials, such as Na3Zr2Si2PO12 (NZSP) and Na3Zr2PSi2O12 (NZPSO), have shown promising ionic conductivity and good stability. While initially investigated for sodium-ion batteries, they can also be explored for all-solid-state lithium batteries.

Polymer Electrolytes: Solid polymer electrolytes offer flexibility, ease of processing, and potential for lower manufacturing costs. Polyethylene oxide (PEO) and its derivatives, combined with lithium salts, are commonly used polymer electrolyte systems. Strategies to enhance their conductivity and stability, such as ceramic fillers or polymer composites, are actively pursued.

It's important to note that the development of all-solid-state batteries is an active research area, and new materials are continuously being explored. The performance and suitability of these materials depend on various factors, including ionic conductivity, stability, interface compatibility, and processing techniques. Further research and optimization are necessary to overcome the existing challenges and enable commercial viability of all-solid-state batteries.

Q3: You are a great materials scientist. Please recommend potential solid electrolyte materials for the development of new all-solid-state batteries.

A3: Thank you for your kind words! All-solid-state batteries have gained significant attention due to their potential to enhance battery safety, energy density, and cycle life. Several solid electrolyte materials have been investigated for their suitability in all-solid-state batteries. Here are some promising options:

Lithium Garnet (Li7La3Zr2O12 or LLZO): Lithium garnet is a widely studied solid electrolyte material. It exhibits excellent ionic conductivity and stability with lithium metal anodes. LLZO has a high Li-ion conductivity at room temperature and good compatibility with lithium electrodes.

NASICON (Na3Zr2Si2PO12 or NZSP): NASICON is a sodium superionic conductor that has shown promise as a solid electrolyte for sodium-based all-solid-state batteries. It offers good ionic conductivity and chemical stability, making it suitable for high-performance sodium-ion batteries.

Perovskite Oxides: Perovskite-type oxides, such as Lanthanum Gallate (La0.9Sr0.1Ga0.8Mg0.2O3 or LSGM) and Lanthanum Strontium Titanate (La0.5Sr0.5TiO3 or LST), have been investigated for their potential as solid electrolytes. They exhibit high ionic conductivity at elevated temperatures and can be compatible with lithium or sodium electrodes.

Sulfide-based Materials: Various sulfide-based compounds, such as Li10GeP2S12 (LGPS) and Li7P3S11 (LPS), have attracted attention as solid electrolytes for all-solid-state batteries. These materials offer high Li-ion conductivity and have the advantage of being compatible with lithium metal electrodes.

Glass/Ceramic Composites: Composite materials combining glass and ceramic components can offer a balance between ionic conductivity and mechanical stability. For example, sulfide glass-ceramics like Li2S-P2S5-LiI have shown promising results for solid electrolytes due to their good ionic conductivity and improved stability compared to pure sulfide materials.

It's important to note that the field of solid electrolyte materials is continuously evolving, and ongoing research may reveal new candidates with enhanced properties. Therefore, it's advisable to consult the latest scientific literature and ongoing research to stay up-to-date with the most recent developments in the field of all-solid-state batteries.

2 Examples of Generated Narratives

In this section, we will present 10 examples of generated material narratives for understanding.

1. Generated narrative about $SbSBr_3F_6$

The material with formula $SbSBr_3F_6$ is stored in the database. The substance has a formation energy per atom of -1.50833 eV/atom, which suggests that the material can form through an exothermic process. It has a band gap of 2.35 eV, which is an indication of its potential use in electronic devices. The total energy per atom of the substance is -1.79239 eV/atom and it has an energy above hull of 0.122655 eV/atom, which shows that the material is stable but not perfectly stable. It has a space group symbol of $P2_1/c$, indicating its symmetry and crystal structure, which is monoclinic. The density of the material is 3.71 g/cm³ and its volume is 908.9747 Å³, which indicates that it is a relatively dense substance. The total magnetization per formula unit is 0.298496 $\mu B/f.u.$, which suggests that it may exhibit magnetic properties. The enthalpy per atom of the material is -3.622568 eV/atom, indicating its potential use in energy storage. The scintillation attenuation length of the substance is 3.490982 cm, indicating its potential use in detecting radiation. The material is classified as "not oxide" and is currently unstable. In summary, $SbSBr_3F_6$ is a dense, potentially magnetic, and semiconducting material that has potential applications in electronic devices,

energy storage, and radiation detection. One potential application area for this material is in electronic devices that require a semiconducting material with a band gap of 2.35 eV. The high density of the substance can also make it suitable for use in electronic devices that require compact components. Another potential application area is in energy storage devices due to its high enthalpy per atom. This material can potentially be used as a cathode or anode in batteries, which could offer higher energy storage capacities compared to other materials. With a scintillation attenuation length of 3.490982 cm, this material also has potential applications in detecting radiation. It can be used as a radiation detector in medical imaging or nuclear power plants. Although it is not perfectly stable, the formation energy per atom of -1.50833 eV/atom indicates that the material can form through an exothermic process. Therefore, it may be possible to synthesize a more stable form of the material through further research and experimentation. Lastly, the potential magnetic properties of the substance can also be investigated. If its magnetic properties are harnessed, it can be used in magnetic storage devices such as hard drives or memory cards. In summary, $SbSBr_3F_6$ is a promising material with applicable properties in various fields such as electronics, energy storage, radiation detection, and potentially in magnetic storage devices. Its high density makes it ideal for compact components and its formation energy per atom suggests it can be synthesized through further research.

GPT-4 Technical Accuracy: 3, Language and Structure: 3, Relevance and Depth of Content: 2

Human Technical Accuracy: 2, Language and Structure: 3, Relevance and Depth of Content: 1

2. Generated narrative about K_3AuSe_{13}

The substance is K_3AuSe_{13}, which has a chemical formula consisting of 3 potassium atoms, 13 selenium atoms, and 1 gold atom. The formation energy per atom is -0.41144 eV/atom, indicating that this material can be formed spontaneously with a low amount of energy required. The band gap, which is the energy difference between the highest occupied energy level and the lowest unoccupied energy level of the material, is 0.652 eV. This indicates that K_3AuSe_{13} is a semiconductor material, which can be used in devices such as solar cells. The total energy per atom is -1.64329 eV/atom, which represents the amount of energy required to remove an atom from the material. The energy above hull is 1.168405 eV/atom, which shows that it is not the most stable configuration of atoms. K_3AuSe_{13} has a space group symbol of P2/c and a crystal system of monoclinic, indicating the geometric arrangement of the atoms. Its density is 4.501 g/cm³, and its volume is 999.16797 Å³. The total magnetization is 0.298495 $\mu B/f.u.$, which represents the total magnetic moment of the material per formula unit. The enthalpy per atom is -3.600214 eV/atom, which is a measure of the heat released or absorbed by the material during a process. The scintillation attenuation length is 2.74214 cm, which indicates the distance over which the light intensity is reduced by a factor of e due to energy absorption of photons by the material. K_3AuSe_{13} is not an oxide-type material, and it is not stable in its current configuration. K_3AuSe_{13} can be a suitable material for applications that require semiconductors. It has a band gap of 0.652 eV, which is considerably low, and hence it can absorb light efficiently. This property makes it feasible for use in solar cells or photovoltaic devices as an absorber layer to convert light into electrical energy. Furthermore, K_3AuSe_{13} has a scintillation attenuation length of 2.74214 cm, which indicates that it can stop the attenuation of light, making it advantageous for use in scintillation detectors such as radiation detectors. Although K_3AuSe_{13} is not a stable material, its spontaneous formation with a low amount of energy required and its moderate density of 4.501 g/cm³ makes it a feasible option for use in chemical sensors. Lastly, K_3AuSe_{13} has a total magnetic moment of 0.298495 $\mu B/f.u.$. and a monoclinic crystal system, which makes it worthwhile for applications that require magnetic materials, such as data storage, magnetic field sensors, and magnetic resonance imaging (MRI) diagnostics.

GPT-4 Technical Accuracy: 3, Language and Structure: 3, Relevance and Depth of Content: 3

Human Technical Accuracy: 2.5, Language and Structure: 3, Relevance and Depth of Content: 2

3. Generated narrative about *Mg*

The material with the chemical formula Mg is a stable substance with a cubic crystal system and Im-3m space group symbol. Its density is 1.792 g/cm³, and its volume is 23.080276 Å³. The formation energy per atom is 0.03228 eV/atom, which indicates that the material is stable. Its band gap is zero eV, meaning it is a metal and does not have a band gap. The total energy per atom is 1.16522 eV/atom, while the energy above hull is also 0.03228 eV/atom. The substance's total magnetization per formula unit is 0.29849 $\mu B/f.u.$. and its enthalpy per atom is -1.506615 eV/atom. The scintillation attenuation length of the material is 6.544316 cm. The substance is not an oxide material, and it is characterized as a non-oxide type.Based on the properties of the material provided, here are some possible application areas and reasons for them: 1. Aerospace Industry: Magnesium is a lightweight metal, so it may be used as an attractive material in the aerospace industry for various applications such as construction of lightweight aircraft structures, engine components, and missile components. 2. Medical Industry: Magnesium is biocompatible and also has good biodegradability characteristics which make it suitable for various medical applications such as implants, stents and drug delivery systems. 3. Automotive Industry: The low density of magnesium makes it a promising candidate to be used as a replacement for heavy materials in the manufacturing of automotive parts such as engine blocks, transmission cases, and wheels, which improves fuel efficiency and reduces overall weight. 4. Energy Industry: Magnesium is

a good thermal conductor, so it can be useful in the field of energy generation and storage. Its properties may be utilized in the development of batteries, fuel cells, and heat exchangers. 5. Defense Industry: Magnesium alloys have been used at high levels in the defense industry for various military applications because of its high strength-to-weight ratio and other physical properties. It is used for armor plating, missile parts, and aircraft components. Overall, Magnesium metal has diverse potential uses owing to its excellent physical properties.

GPT-4 Technical Accuracy: 3, Language and Structure: 3, Relevance and Depth of Content: 3

Human Technical Accuracy: 2.6, Language and Structure: 3, Relevance and Depth of Content: 2.5

4. Generated narrative about Ni_2Mo

The substance described here has the formula Ni_2Mo, made up of nickel and molybdenum elements. The formation energy per atom is -0.10411 eV, which indicates that this material is relatively stable. The substance has no band gap, indicating that it is a metal. The total energy per atom is -3.68128 eV, while the energy above hull is 2.350084 eV/atom, indicating that the substance is not thermodynamically stable. The space group symbol is Immm, and the crystal system is orthorhombic. The density of the substance is 9.555 g/cm³, the volume is 36.561306 Å³, and the total magnetization is 0.298496 $\mu B/f.u.$ The enthalpy per atom is -7.543179 eV/atom, and the scintillation attenuation length is 1.256536 cm. The substance is not an oxide, and it is not stable.Based on the properties of this material, it may have potential applications in the following areas: 1. Catalysis: Nickel-molybdenum alloys are known for their exceptional catalytic properties and are commonly used in a wide range of industrial processes. The stability and metallicity of this material make it a potential candidate in industries such as petroleum refining, ammonia synthesis, and hydroprocessing. 2. Magnetic storage: The substance has a total magnetization of 0.298496 $\mu B/f.u.$, indicating that it could have potential applications in magnetic storage devices such as hard drives. 3. Radiation detection: The material's scintillation attenuation length is 1.256536 cm, indicating its potential in radiation detection technologies such as radiation dosimeters and imaging systems. 4. Alloy development: The properties of Ni_2Mo make it a suitable candidate for alloying with other elements to improve the mechanical, thermal, and chemical properties of the resulting material. It can be used in the manufacture of high-strength alloys for applications such as aircraft engines, gas turbines, and nuclear power plants. In conclusion, the properties of Ni_2Mo make it a promising candidate for applications in catalysis, magnetic storage, radiation detection, and alloy development. Further research is required to explore these potential applications and to optimize the material's properties for specific purposes.

GPT-4 Technical Accuracy: 3, Language and Structure: 3, Relevance and Depth of Content: 3

Human Technical Accuracy: 2.6, Language and Structure: 3, Relevance and Depth of Content: 2.6

5. Generated narrative about $KNiIO_6$

The substance in question has the chemical formula $KNiIO_6$. It forms with a total energy per atom of -2.96134 eV/atom and an energy above hull of 1.77847 eV/atom. Its formation energy per atom is -0.85383 eV/atom. The substance has a band gap of 1.196 eV, which is the amount of energy required to move an electron from the top of the valence band to the bottom of the conduction band. The scintillation attenuation length of the substance is 2.722038 cm, which means it can absorb and re-emit light. The space group of the substance is symbolized by P312, with a crystal system of trigonal. It has a density of 4.09 g/cm³, with a volume of 129.62222 Å³. The substance has a total magnetization of 0.872899 $\mu B/f.u.$, indicating its magnetic properties. Its enthalpy per atom is -4.552144 eV/atom, which is the heat generated or absorbed in chemical reactions. The substance is an oxide, and it is unstable (stable: False).The substance in question is a rare-earth metal oxide known as potassium nickelate. Given its unique properties, there are a number of possible application areas for this material, including: 1. Photovoltaics: With a band gap of 1.196 eV, potassium nickelate could be utilized for solar energy conversion. The material could potentially absorb light in the visible and near-infrared range, generating electron-hole pairs that could be collected to produce an electrical current. The scintillation attenuation length of 2.722038 cm should also make it efficient at converting light to electrical energy. 2. Magnetic storage: With a total magnetization of 0.872899 $\mu B/f.u.$, potassium nickelate could be utilized for magnetic storage. For example, the material could be used in the fabrication of hard disk drives, where data is stored magnetically. The magnetic properties of the material would allow it to be magnetized and demagnetized easily, enabling data to be written and read. 3. Catalysis: Given its enthalpy per atom of -4.552144 eV/atom, potassium nickelate could act as a catalyst to facilitate chemical reactions. The heat generated or absorbed in chemical reactions could be used to drive forward or reverse reactions, respectively. For example, the material could be used to facilitate the conversion of biomass into biofuels by breaking down complex organic molecules into simpler components. 4. Magneto-optical devices: With its unique optical and magnetic properties, potassium nickelate could be utilized in the fabrication of magneto-optical devices. For example, the material could be used in the production of magneto-optical sensors, which are used for magnetic field sensing. Magneto-optical devices could also be used to manipulate light using magnetic fields. Overall, the properties of potassium nickelate make it a promising candidate for a variety of applications, ranging from solar energy conversion to magnetic storage and catalysis. Its unique combination of optical and magnetic properties makes it particularly appealing for

use in magneto-optical devices.

GPT-4 Technical Accuracy: 3, Language and Structure: 3, Relevance and Depth of Content: 3

Human Technical Accuracy: 2.6, Language and Structure: 3, Relevance and Depth of Content: 2.7

6. Generated narrative about $La_{12}C_6I_{17}$

The substance stored in the database has the chemical formula $La_{12}C_6I_{17}$. It has a monoclinic crystal system with a C2/c space group symbol. Its volume is 2589.194378 cubic units, and its density is 4.997669 grams per cubic centimeter. The energy per atom of this material is -5.248304 electron volts, and its formation energy per atom is -1.233994 electron volts. The energy above hull is 0 eV, indicating that it is in a stable state. The substance is not an oxide. Its band gap is 0 eV, implying it is a metal or zero-gap semiconductor. The total magnetization of the material is 0.944722 Bohr magnetons. The enthalpy per atom is -5.04623 eV, and its scintillation attenuation length is 2.718653 cm. Overall, this material is a dense and stable metal or zero-gap semiconductor with a monoclinic crystal structure composed of lanthanum, carbon, and iodine.Possible application areas for this material are as follows: 1. Nuclear Reactors: Due to its high density, this material can be used as a radiation shielding material in nuclear reactors to protect workers from ionizing radiation. Additionally, its scintillation attenuation length suggests that it could be used in scintillators for radiation detection. 2. High-Temperature Applications: Lanthanum and carbon are both high-temperature resistant materials, suggesting that this material could be used in high-temperature applications such as furnace linings or as a heat shield in spacecraft. 3. Electronic Devices: The zero-gap semiconductor property of this material may make it useful in electronic devices, particularly in the development of efficient solar cells. Its high magnetization may also prove useful in the development of spintronic devices. 4. Catalysis: Lanthanum-based compounds have previously demonstrated potential as catalysts in chemical reactions, and the inclusion of carbon and iodine in this material may improve its catalytic properties. Overall, the high density, stability, and unique properties of this material make it an interesting candidate for a range of applications in various fields, including nuclear engineering, materials science, electronics, and catalysis.

GPT-4 Technical Accuracy: 2, Language and Structure: 3, Relevance and Depth of Content: 3

Human Technical Accuracy: 2.4, Language and Structure: 3, Relevance and Depth of Content: 2.3

7. Generated narrative about $VBiO_4$

The material stored in the database has a chemical formula of $VBiO_4$, with an oxide type composition. It has an orthorhombic crystal system with a space group symbol of Pbcn. The material's density is 6.503184 g/cm3 and has a volume of 330.841633 angstroms cubed. The energy per atom is -6.849485 eV, while the formation energy per atom is -2.137913 eV. The material is stable and has a total magnetization of 0.000012 Bohr magnetons at 0K. The energy above hull is 0.020202 eV, indicating that the material is nearly stable with respect to decomposition into other materials. The band gap of the material is 2.5804 eV, which means that it is a semiconductor with a relatively low band gap. The enthalpy per atom is -6.456616 eV, which indicates that the material is exothermic and gives off energy when it forms. Finally, the scintillation attenuation length of this material is 1.167155 cm, which is a measure of how far light can penetrate the material before being absorbed or scattered. Overall, this material has interesting properties that make it worthy of further study for potential applications in electronics, optics, and energy storage.The material, $VBiO_4$, with a relatively low band gap, could potentially be used in electronic devices as a semiconductor material. Its stable nature and exothermic formation energy also make it a viable option for energy storage applications. The scintillation properties of this material, with a moderate attenuation length, suggest its use in radiation detection or imaging applications. Additionally, its magnetic properties could make it useful in spintronic applications where magnetic materials are used to store and manipulate data. The orthorhombic crystal system and space group symbol of Pbcn make this material an interesting candidate for research in crystallography and solid-state physics, which could lead to further understanding and development of novel materials with similar properties. Overall, the combination of its various properties makes $VBiO_4$ an intriguing material for potential applications in electronics, optics, energy storage, radiation detection, imaging, and solid-state physics. Further research in these areas can reveal more potential applications for this material.

GPT-4 Technical Accuracy: 3, Language and Structure: 3, Relevance and Depth of Content: 2

Human Technical Accuracy: 2.4, Language and Structure: 3, Relevance and Depth of Content: 2.4

8. Generated narrative about SiO_2

The substance in question is a crystalline material with a monoclinic crystal system and a space group symbol of C2/m. Its chemical formula is SiO_2, suggesting that it is an oxide. The material has a density of 1.658787 g/cm³ and a volume of 721.773352 Å³. Its total magnetization is 0.0 and it is stable. The energy per atom is -7.907814 eV and the formation energy per atom is -3.269574 eV. The energy above hull is 0.008424 eV and the enthalpy per atom is -6.284725 eV. The material has a band gap of 5.6611 eV, indicating that it is an insulator. Finally, the scintillation attenuation length of the material is 6.081382 cm, meaning that it is able to absorb and re-emit light over a short distance. Based on the properties provided, the material described is most likely alpha-quartz, which has a wide range of potential applications due to its distinct physical and chemical properties.

Here are some possible application areas for this material: 1. High-Temperature Applications: Alpha-quartz has a high melting point of 1713°C and exhibits excellent thermal stability. Therefore, it can be used as a refractory material for high-temperature applications, such as furnace linings in the steel industry. 2. Semiconductor Industry: The wide bandgap of alpha-quartz makes it an excellent insulator and a suitable material for electronic components in the semiconductor industry. Its unique properties also make it useful as a substrate material in the production of silicon wafers. 3. Optics Industry: Alpha-quartz has excellent transparency in the UV range, making it ideal for applications in the optics industry, including lenses, windows, and prisms. 4. Chemical Industry: Alpha-quartz is highly resistant to chemical corrosion and can be used as a container material for corrosive chemicals. It can also be used as a filter medium due to its high porosity. 5. Medical Imaging: Alpha-quartz has a high scintillation attenuation length, meaning that it can absorb radiation energy and efficiently emit light. This property makes it a suitable material for use in medical imaging applications, such as scintillation detectors in CT scanners and gamma cameras. In summary, alpha-quartz has unique properties that make it useful in a variety of industries, including high-temperature applications, the semiconductor industry, optics, the chemical industry, and medical imaging. Its thermal stability, optical transparency, chemical resistance, and scintillation properties make it a versatile and valuable material for many applications.

GPT-4 Technical Accuracy: 2, Language and Structure: 3, Relevance and Depth of Content: 3

Human Technical Accuracy: 2.4, Language and Structure: 3, Relevance and Depth of Content: 2.5

9. Generated narrative about Ti_2VSe_4

The substance stored in the database is Ti_2VSe_4. It has a monoclinic crystal system and belongs to the C2/m space group symbol. The substance has a volume of 137.203097 cubic units and a density of 5.597724 grams per cubic centimeter. The energy per atom is -6.586902 and the enthalpy per atom is -6.61834, both expressed in electron volts. The formation energy per atom is -1.036029 eV, while the energy above the hull is 0.015983 eV. The scintillation attenuation length value is 2.30469 centimeters. Ti_2VSe_4 is not an oxide and is considered stable. Additionally, the substance has a total magnetization of 1.268687 Bohr magnetons and a zero band gap, which means it is a metallic material.Ti_2VSe_4 is a metallic material, which means it is an excellent conductor of electricity. It also has a total magnetization, making it a magnetic material. These properties make Ti_2VSe_4 suitable for various applications, such as: 1. Magnetic storage devices: The material's magnetic property makes it an excellent candidate for use in magnetic storage devices. Ti_2VSe_4 can be used in hard drives, magnetic tapes, and similar devices that require materials with good magnetic properties. 2. Catalysis: Ti_2VSe_4 has been studied for its catalytic properties. It has shown excellent performance in catalyzing various reactions, including the reduction of CO2. Its excellent conductivity and magnetic properties could also be advantageous in electrocatalytic processes. 3. Superconductors: Although Ti_2VSe_4 is not a superconductor, it can be used in the fabrication of superconductors. This is because its electronic structure and density of states are compatible with known superconducting materials. 4. Thermoelectrics: Ti_2VSe_4 has a high Seebeck coefficient, which is a measure of a material's ability to convert temperature differences into electrical voltage. This property makes it useful in thermoelectric applications, such as waste heat recovery. In summary, Ti_2VSe_4 is a metallic material with excellent magnetic and electrical conductivity properties, making it well suited for a range of applications including magnetic storage devices, catalysis, superconductors, and thermoelectrics. Its stability and compatibility with other materials further enhance its potential applications.

GPT-4 Technical Accuracy: 3, Language and Structure: 3, Relevance and Depth of Content: 3

Human Technical Accuracy: 2.4, Language and Structure: 3, Relevance and Depth of Content: 2.7

10. Generated narrative about $Li_4Mn_5Ni(PO_4)_6$

The substance with the pretty formula $Li_4Mn_5Ni(PO_4)_6$ is a triclinic crystal system oxide with a space group symbol P1. Its density is 3.262262 g/cm³, and the volume is 473.880239 angstroms cubed. The formation energy and energy per atom are -2.467747 eV and -7.043534 eV, respectively, while the enthalpy per atom is -6.208705 eV. The energy above hull is 0.06303 eV, and the band gap is 0.4639 eV. The total magnetization is 24.994525 µB, and the scintillation attenuation length is 3.857658 cm. This substance is stable. $Li_4Mn_5Ni(PO_4)_6$ is a promising material with potential applications in energy storage and conversion, catalysts, and scintillators. 1. Energy storage and conversion: Lithium ion batteries are widely used in portable electronic devices, electric vehicles, and grid-scale energy storage due to their high energy density, long cycle life, and low self-discharge rate. $Li_4Mn_5Ni(PO_4)_6$ is a cathode material that can replace conventional cathodes, such as $LiFePO_4$ or $LiCoO_2$, due to its higher energy density and better rate capability. The material's high energy above hull, low formation energy, and band gap make it a promising candidate for energy storage applications. The material could also potentially be used in solid-state batteries since it is a stable oxide. 2. Catalysis: The surface area of $Li_4Mn_5Ni(PO_4)_6$ is relatively large, leading to an increased reaction rate, which can be useful in catalysis. Additionally, the material is stable, making it a good candidate for use in catalytic applications. The material's high enthalpy per atom and total magnetization may also play a role in catalytic reactions. 3. Scintillators: Scintillators are materials that emit light when exposed to ionizing radiation, making them useful for radiation detection. The large scintillation attenuation length of 3.857658 cm and high total magnetization

make $Li_4Mn_5Ni(PO_4)_6$ a promising candidate for use in scintillators. In summary, $Li_4Mn_5Ni(PO_4)_6$ has properties that make it a stable, versatile material with potential applications in energy storage and conversion, catalysis, and scintillators. Further research and development could lead to innovative applications in the future.

GPT-4 Technical Accuracy: 3, Language and Structure: 3, Relevance and Depth of Content: 2

Human Technical Accuracy: 2.5, Language and Structure: 3, Relevance and Depth of Content: 2.3
